# Study on the influence of trauma-informed music intervention on emotional adjustment and social adaptability of children exposed to pediatric trauma

**DOI:** 10.3389/fpubh.2026.1884440

**Published:** 2026-07-22

**Authors:** Xieping Chen, Qian Xie

**Affiliations:** School of Educational Science, Leshan Normal University, Leshan, Sichuan, China

**Keywords:** children's psychological rehabilitation, emotional regulation, pediatric trauma exposure, social adaptation, trauma-informed music intervention

## Abstract

**Background:**

Children exposed to traumatic events frequently develop emotion dysregulation and impaired social adaptation, which severely impairs their psychosocial development. As a non-invasive, well-tolerated psychological intervention, trauma-informed music intervention yields promising therapeutic gains for traumatized children's emotional and social functioning. Nevertheless, high-quality quantitative clinical trials remain lacking to corroborate its clinical efficacy.

**Methods:**

This randomized controlled trial recruited 120 trauma-exposed children hospitalized in our institution between January 2024 and June 2025, randomly allocated to an intervention group (*n* = 60) and a control group (*n* = 60). The control group received standard psychological care only. The intervention group received an additional 8-week trauma-informed music intervention (twice weekly, 45 min per session), consisting of structured modules including musical improvisation, rhythmic training and emotion-oriented song creation. Outcome assessments were conducted at three time points (pre-intervention, post-intervention, 3-months follow-up) using the Children's Emotion Regulation Questionnaire (ERQ-C), Social Skills Improvement System (SSIS), and Screen for Child Anxiety Related Emotional Disorders (SCARED). Respiratory sinus arrhythmia (RSA) was measured as a physiological biomarker of autonomic regulatory function.

**Results:**

At post-intervention, the intervention group demonstrated substantially higher ERQ-C total scores (48.72 ± 6.35 vs. 35.18 ± 7.24) and SSIS total social skills scores (52.41 ± 7.03 vs. 39.27 ± 6.89) relative to the control group (*P* < 0.001), alongside marked reductions in SCARED anxiety-depression scores (22.36 ± 5.17 vs. 38.65, *P* < 0.001). The cooperation, empathy and social initiative subscales of the SSIS reflected the largest improvements. The intervention group displayed significant elevations in RSA magnitude (*P* < 0.01), signifying upregulated parasympathetic nervous system activity. All intervention-related gains persisted stably at the 3-months follow-up, with Cohen's *d* ranging from 0.78 to 0.92. No severe adverse events were recorded during the entire trial period.

**Conclusion:**

Trauma-informed music intervention improves emotion regulation and social functioning, and mitigates internalizing symptoms among children with pediatric trauma exposure. The intervention delivers durable therapeutic effects with satisfactory safety profiles, and can serve as a valid non-pharmacological adjunct for psychological rehabilitation of traumatized children.

## 1 Introduction

Childhood trauma exposure (including maltreatment, neglect, witnessing violence and catastrophic disasters) represents a critical global public health issue. Traumatic events alter structural and functional brain architecture in children and induce emotion dysregulation, aberrant stress reactivity and social adaptive deficits. In severe cases, trauma sequelae persist into adulthood, elevating risks of anxiety, depression and post-traumatic stress disorder (PTSD) and exerting long-term adverse impacts across the lifespan (1). Neuroimaging evidence verifies that childhood trauma disrupts core brain regions including the prefrontal cortex, amygdala and hippocampus, and triggers hypothalamic–pituitary–adrenal (HPA) axis dysregulation. These neurological alterations drive hallmark trauma-related manifestations: diminished emotion regulation capacity, heightened negative emotional reactivity and social withdrawal, constituting a leading risk factor for childhood psychological and behavioral dysfunction (2). Epidemiological data indicate 22%−35% of children worldwide experience at least one traumatic event. School-age children (7–12 years) are disproportionately vulnerable to traumatic stress amid rapid cognitive and social maturation, with severe impairments to regulatory and social functioning. This underscores an urgent demand for early, safe and effective intervention protocols (3).

Trauma-Focused Cognitive Behavioral Therapy (TF-CBT) and Eye Movement Desensitization and Reprocessing (EMDR) remain the gold-standard psychotherapies for traumatized children. While their therapeutic efficacy is well-established, these approaches carry notable drawbacks: prolonged delivery cycles, limited specialized clinicians, low child treatment adherence, and heavy reliance on verbal expression capacity, hindering large-scale clinical dissemination (4). For traumatized children with limited verbal skills and severe emotional dysregulation, conventional talk-based therapies fail to access underlying affective experiences, resulting in muted intervention gains (5). Accordingly, developing novel non-invasive, child-friendly, easy-to-administer interventions with high acceptability has emerged as a prominent research priority.

As an important branch of art therapy, trauma-informed music intervention is grounded in neuroscience, psychology and traumatology theories. Through structured musical activities including improvisation, rhythmic exercises and emotional song creation, it offers traumatized children a safe, low-pressure outlet for emotional expression. Bypassing linguistic barriers and directly targeting the limbic system and autonomic nervous system, it is hypothesized to modulate stress responses and facilitate emotional integration and social connection (6, 7). Studies suggest that music intervention can significantly lower children's cortisol levels, boost parasympathetic activity and optimize physiological indicators such as respiratory sinus arrhythmia (RSA), which lays a physiological foundation for emotional regulation (8). Meanwhile, group musical activities cultivate children's sense of cooperation and empathy, stimulate their social initiative, reduce social avoidance and loneliness, and ultimately improve their social adaptability (9). In recent years, a number of overseas randomized controlled trials have verified that trauma-informed music intervention can effectively relieve anxiety and depressive symptoms and improve emotional regulation and social functioning in traumatized children, with high safety and no adverse events observed (10).

Trauma-informed music intervention exhibits great potential in the psychological rehabilitation of traumatized children. Nevertheless, existing studies have methodological flaws including small sample sizes, inconsistent intervention protocols, short follow-up periods, and a lack of multi-center, large-sample randomized controlled trials. Its improving effects and quantitative evidence regarding emotional regulation and social adaptability among school-age children with trauma exposure still require further verification via high-quality clinical research. Meanwhile, most previous studies fail to separate the confounding effects generated by extra interpersonal care and group activities from the independent effects of music intervention, and their mechanistic discussions are mostly limited to theoretical speculation. Therefore, this study thoroughly explores the impacts of trauma-informed music intervention on emotional regulation and social adaptability in children with pediatric trauma exposure, analyzes the preliminary characteristics of intervention effects, and distinguishes statistical differences from clinical value. It aims to provide novel and effective intervention strategies as well as scientific evidence for the psychological rehabilitation of traumatized children, and advance the clinical application and popularization of trauma-informed music intervention in the domestic child mental health field.

## 2 Materials and methods

### 2.1 Data collection and processing of trauma sample information

#### 2.1.1 Source of participants

**Figure 1:**
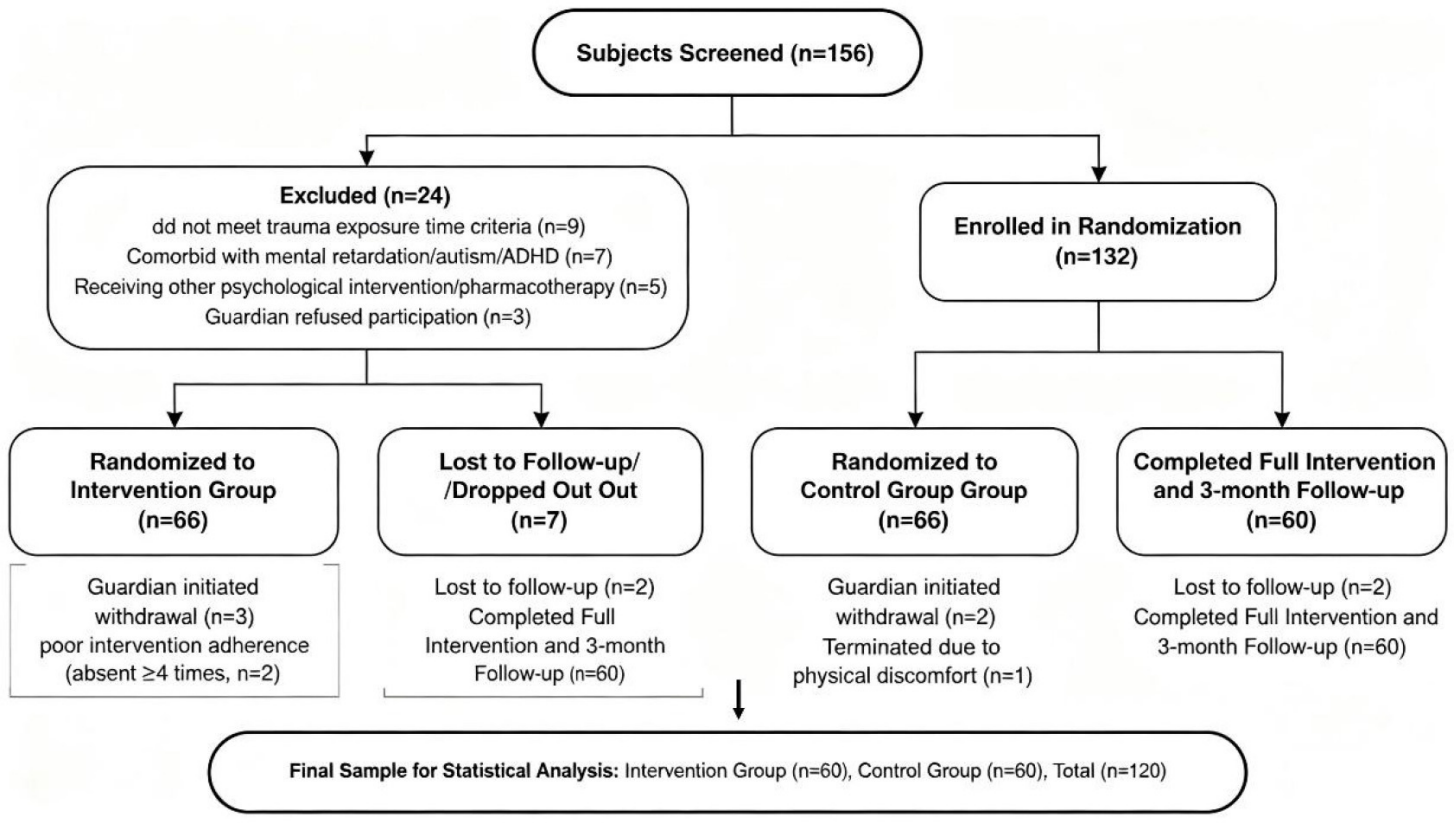
CONSORT flow diagram.

Children with traumatic exposure admitted to the pediatric outpatient department and hospital in our hospital from January 2024 to June 2025 were selected as the research subjects. All the research subjects were confirmed by the joint evaluation of pediatricians and psychotherapists in our hospital, which met the diagnostic criteria of traumatic exposure and the exclusion criteria of this study. This study was approved by the Medical Ethics Committee of our hospital, and the research scheme followed the ethical principles of Helsinki Declaration. All guardians of the research subjects signed written informed consent forms, and for children aged 7 and above who have basic understanding ability, additional informed consent forms were signed; The researcher has fully informed them of the research purpose, intervention process, potential risks and benefits, and all the subjects volunteered to participate in this study and promised to cooperate with them to complete the whole evaluation and follow-up. This study was a randomized controlled clinical trial registered with the Chinese Clinical Trial Registry. The whole process of subject screening, enrollment, randomization and follow-up was conducted in strict accordance with the standard CONSORT participant flow diagram (see Figure 1).

#### 2.1.2 Inclusion criteria

① Age 6–12 years old, regardless of sex, able to understand simple instructions normally, communicate with basic language, and cooperate with the completion of scale evaluation and music intervention; ② Clear trauma exposure history confirmed by clinical evaluation, including but not limited to accidents (traffic crashes, falls, burns), violent injury, bereavement and sudden disasters, with trauma exposure time within 1–6 months; ③ The score of SCARED is ≥25, which indicates that there are mild and above anxiety and depression symptoms; ④ The total score of Children's Emotional Regulation Scale (ERQ-C) is ≤ 40, suggesting that there is emotional regulation disorder; ⑤ The guardian and the child live together for a long time, and can cooperate to complete the intervention arrangement and follow-up, without serious mental illness, cognitive disorder and communication disorder.

#### 2.1.3 Exclusion criteria

① There are serious organic diseases (such as congenital heart disease, nervous system diseases, severe liver and renal insufficiency, etc.), and music intervention cannot be tolerated; ② Intellectual disability, autism spectrum disorder, attention deficit hyperactivity disorder and other mental and psychological diseases, which affect the evaluation of emotional and social functions; ③ Being receiving other psychological intervention (such as cognitive behavioral therapy, sand table therapy) or psychiatric medication, and unable to pause; ④ There is obvious resistance to music intervention, or the guardian does not cooperate with the intervention and follow-up; ⑤ Trauma exposure time is more than 6 months, or has received systematic trauma rehabilitation intervention; ⑥ During the follow-up, the whole evaluation cannot be completed, or the intervention needs to be terminated if there are serious adverse reactions.

#### 2.1.4 Sample size estimation

Sample size calculation was performed via G^*^Power 3.1, referencing comparable trauma intervention trials. The ERQ-C total score was designated as the primary outcome measure, with a two-tailed α = 0.05, β = 0.10, and hypothesized Cohen's *d* = 0.40. The analysis yielded a target effective sample of 120 participants. Accounting for an anticipated 10% attrition rate among child participants, 132 individuals were initially recruited. Twelve participants withdrew throughout the trial (7 from the intervention group, 5 from the control group). The final analytic sample comprised 120 children evenly split into intervention (*n* = 60) and control (*n* = 60) arms, satisfying pre-specified statistical power requirements.

#### 2.1.5 Dropout criteria

① Serious adverse reactions (such as violent emotional fluctuation and physical discomfort) occurred during the intervention, and the intervention needs to be terminated after evaluation; ② The guardian voluntarily withdrew from the study, or could not continue to participate because of relocation, medical treatment and other reasons; ③ Lost contact during the follow-up period, unable to complete the evaluation after intervention and follow-up for 3 months; ④ The compliance of intervention was poor, and the cumulative number of absent interventions was ≥4 times (the total number of interventions was 16 times), or they did not cooperate with the scale evaluation and physiological index detection.

### 2.2 Research design and grouping method

This study adopted a randomized controlled trial design following the principles of randomization, control and single blinding of outcome assessors. A random number table was used to generate random sequences ranging from 1 to 12 independently by third-party statisticians. Participants were assigned sequentially according to their enrollment order: odd numbers were allocated to the intervention group and even numbers to the control group, with 60 cases in each group. Sequentially numbered, sealed opaque envelopes were utilized to ensure allocation concealment. Given the obvious differences in intervention modes between the two groups, blinding of intervention implementers, participants and their guardians was not feasible. Only outcome evaluators and statistical analysts remained blinded throughout the whole study to reduce assessment bias. After grouping, balanced comparison of general data (age, gender, trauma type, trauma exposure duration, family structure, parental educational attainment) and all pre-intervention assessment indicators (ERQ-C, SSIS, SCARED scores and RSA levels) was carried out between the two groups to guarantee comparable baseline data (*P* > 0.05).

### 2.3 Intervention measures

The subjects in both groups received routine pediatric nursing care and routine psychological support with the same frequency and duration. In the intervention group, 45 min of structured music intervention was added twice a week. Compared with the control group, the intervention group gained more opportunities for therapists' contact time, interpersonal interaction and group activities, all of which were potential confounding variables. In the intervention group, 8 weeks of trauma-informed music intervention was added, while in the control group, only routine intervention was maintained, without any additional music-related intervention measures. During the intervention period and follow-up period, no other psychological intervention or psychiatric medication was accepted.

#### 2.3.1 Routine psychological support (for both groups)

Implemented by full-time psychotherapists in our hospital, once a week for 30 min, lasting for 8 weeks. The main contents include: emotional comfort, trauma-related cognitive counseling, parent-child communication guidance, relaxation training (such as deep breathing and gradual muscle relaxation), helping children and guardians to relieve the negative emotions caused by trauma, understand the normal performance of psychological reaction after trauma, and master the basic emotional adjustment methods.

#### 2.3.2 Trauma-informed music intervention (exclusive to intervention group)

It is implemented by professionals who have the qualification of music therapist (holding the professional qualification certificate of national music therapist) and have more than 5 years of experience in pediatric psychological intervention. The intervention place is the music therapy room specially set up in our hospital (quiet and comfortable, equipped with sound insulation equipment, piano, African drum, xylophone, sand hammer, hand bell and other musical instruments, with soft light to avoid external interference).

Intervention cycle: 8 weeks, twice a week, 45 min each time, with an interval of 3–4 days, with a total of 16 interventions, and each intervention time is fixed at 9:00–9:45 am or 15:00–15:45 pm to avoid affecting children's normal work and rest and treatment. The intervention attendance of each child was recorded throughout this study, and the overall compliance rate of intervention attendance is detailed below.

Intervention modules and specific processes (each intervention follows the following structured modules and is promoted step by step):

(1) Relationship-building stage (weeks 1–2): 5 min before each intervention, have a relaxed interaction with children (such as musical instrument touch and simple rhythm imitation), establish a trust relationship, explain the intervention objectives and procedures to children to reduce treatment resistance; The core module is “rhythm rhythm”, which adopts simple and easy-to-learn rhythms (such as 2/4 beat and 4/4 beat), so that children can use simple musical instruments such as sand hammer and hand bell to follow the music, support children to externalize emotions and process unresolved trauma-related negative affect over 15 min per session; 5 min before the end, make a brief summary, encourage children to share their feelings of participation, and strengthen the positive experience.
(2) Emotional expression stage (3–5 weeks): Continue the rhythm module (10 min), increase the rhythm complexity, and guide children to express different emotions (such as tension, sadness, happiness) through the pace and intensity; The core module is “Emotional Song Creation” (20 min), which combines children's traumatic experience and emotional state, guides children to create simple lyrics (1–2 sentences) with familiar melodies (such as children's ballads adaptation), encourages children to sing loudly, releases their negative emotions and accepts their own feelings; 5 min before the end, conduct emotional combing to help children identify their emotions and establish the relationship between emotions and music expression.
(3) Skills upgrading and integration stage (weeks 6–8): rhythm module (10 min), joining group interactive rhythm games to cultivate children's sense of cooperation; The core module is “improvisation” (20 min), which allows children to choose musical instruments freely, improvise according to their own emotions, encourage children to express boldly and improve the flexibility of emotional adjustment; Add a “social interaction” sub-module (10 min) to cultivate children's empathy, social initiative and cooperation ability through the cooperation of two musical instruments and group ensemble; 5 min before the end, review the whole intervention process, strengthen children's positive changes, and guide children to apply the emotional adjustment and social skills learned in the intervention to their daily lives.

Intervention quality control: formulate a unified operation manual for trauma-informed music intervention, and clarify the operation standards, time allocation and precautions of each module; Intervention implementers shall conduct training and case discussion once a week to ensure standardized intervention process and unified operation; Assign a special person to record the whole process of intervention, including children's participation, emotional reaction and cooperation, and check the compliance of intervention every week. For children who are absent from intervention, communicate with their guardians in time and arrange supplementary intervention (supplementary intervention should be completed within 24 h after the original intervention time, and the number of supplementary interventions should not exceed 2 times).

### 2.4 Outcome measures and assessment time points

All the subjects completed the testing of the following evaluation indicators before intervention (T0), immediately after intervention (T1) and after 3 months of follow-up (T2). After unified training, the evaluators strictly followed the scale and physiological indicators testing standards to ensure the accuracy and consistency of the evaluation results. The follow-up period of this study is only 3 months, and the trauma-related symptoms have the characteristics of chronic delay, which is not enough to judge the long-term and long-term curative effect of intervention.

#### 2.4.1 Main evaluation indicators

(1) Emotion regulation ability: The children's emotion regulation questionnaire for children (ERQ-C) was adopted, which was compiled by Gross et al. and revised by domestic scholars for children aged 6–12, with good reliability and validity (Cronbach's α coefficient is 0.82–0.86). There are 10 items in the scale, which are divided into two dimensions: cognitive reappraisal (6 items) and expression inhibition (4 items). The scale uses a five-point scoring method (1= completely inconsistent, 5= completely consistent), and the total score ranges from 10 to 50. Higher total scores correspond to superior emotion regulation capacity of children. During the evaluation, the evaluators explain the meaning of the items to the children one by one, and the children answer according to their own actual situation, accompanied by guardians, without interfering with the children's answers.
(2) Social adaptability: Social Skills Improvement System (SSIS) is adopted, which is suitable for children aged 6–12. The revised Cronbach's α coefficient is 0.80–0.88, with good reliability and validity. There are 32 items in the scale, which are divided into four dimensions: cooperation, empathy, social initiative and self-control. The scale adopts a four-point scoring method (1= almost never, 4= almost always), and the total score ranges from 32 to 128. The higher the total score and the scores in each dimension, the better the children's social adaptability and social skills. The scale was filled out by the guardian according to the children's performance in the past week. As caregivers were unblinded to group assignment and intervention schedules, caregiver-reported SSIS outcomes are subject to response bias, warranting cautious interpretation of social skills findings. The evaluation is answered by the guardian according to the actual performance of the children in the past week, and the evaluators explain the items of the scale to ensure that the guardian understands the meaning of the items before filling them in.

#### 2.4.2 Secondary evaluation indicators

(1) Internalized symptoms (anxiety and depression): Screen for Child Anxiety Related Emotional Disorders (SCARED) is adopted, which is a commonly used scale for screening anxiety and depression in children, and is suitable for children aged 6–18. The Cronbach's α coefficient of the revised version is 0.85–0.90, and its reliability and validity are reliable. There are 41 items in the scale, which are divided into two dimensions: anxiety (37 items) and depression (4 items). The scale uses a three-point scoring method (0 = none, 1 = sometimes, 2 = often), and the total score ranges from 0 to 82. The total score ≥25 is mild anxiety and depression, and ≥40 is moderate and above anxiety and depression. The lower the total score, the lower the anxiety and depression symptoms of children. During the evaluation, the appraisers guide the children to answer according to their own feelings, and the younger children (6–8 years old) can be assisted by their guardians to understand the items, but they do not interfere with the answer results.
(2) Autonomic nerve regulation function: Detecting Respiratory Sinus Arrhythmia (RSA) as a physiological index of emotional regulation. Multi-channel physiological recorder was used, operated by professional medical staff, and the detection environment was quiet. When children were seated and relaxed, ECG signals were recorded for 5 min, and the sampling frequency was 1,000 Hz. RSA index was extracted by physiological signal analysis software, mainly focusing on the high-frequency components of RSA (0.15–0.40 Hz), and RSA value (unit: ms^2^) was used as the evaluation index. RSA can reflect parasympathetic nervous system activity to a certain extent, but it is easily disturbed by many factors such as respiratory rhythm, emotional fluctuation and environmental stimulation. It is impossible to directly confirm the improvement of emotional regulation function and deduce the specific neurophysiological mechanism only by RSA. The higher the RSA value, the stronger the parasympathetic nervous system activity was. Before testing, inform the children and their guardians of the testing process to eliminate nervousness, ensure that children remain quiet and relaxed during testing, and avoid interference factors such as strenuous activities and crying.

#### 2.4.3 Evaluation time node

(1) T0: within 1 week before the intervention, complete all scale evaluation and RSA detection, and collect the general data and baseline index data of the research object;
(2) T1: After 8 weeks of intervention, complete all scale evaluation and RSA detection within 24 h, and record the changes of indicators after intervention;
(3) T2: 3 months after the intervention, all scales and RSA tests were completed through outpatient follow-up or telephone follow-up to evaluate the durability of the intervention effect.

During the monitoring and intervention of adverse reactions, after each intervention, the intervention personnel will observe the children's emotional state and physical reactions (such as headache, nausea, crying, etc.), and record the occurrence of adverse reactions (type, occurrence time, severity, treatment method and outcome); During the follow-up period, the children's adverse reactions related to the intervention were monitored through the feedback of guardians to ensure the safety of the intervention.

### 2.5 Statistical methods

SPSS 26.0 was used for data sorting and analysis. Shapiro-Wilk test and Levene's variance homogeneity test were performed for all data. This study is a randomized controlled trial of repeated measurement, combined with multi-outcome index and multi-time point design, and mainly uses mixed effect model and repeated measurement variance analysis with interaction items to analyze the interaction effects between groups, within groups and time; χ^2^ test was used for classified data. Continuous data were expressed as mean ± standard deviation. Paired *t*-tests were used for intra-group comparisons before intervention, after intervention and during follow-up; independent sample *t*-tests were adopted for inter-group comparisons at the same time point; repeated measures analysis of variance was applied for intra-group comparisons across multiple time points (T0, T1 and T2). The counting data were expressed by the number of cases (percentage) [*n* (%)], and the comparison between groups was made by χ^2^ test. Pearson correlation analysis was used for correlation analysis; Cohen's *d* (effect size) thresholds were defined as large (*d* ≥ 0.8), medium (0.5 ≤ *d* < 0.8), and small (0.2 ≤ *d* < 0.5). The difference between *P* < 0.05 and *P* < 0.01 was statistically significant. Data quality control arranges a special person to be responsible for data entry, and after the entry is completed, double-check it, and correct it in time when errors are found; The missing data are supplemented by multiple interpolation (missing rate ≤ 5%); For the abnormal values, Grubbs test is used to identify them, and whether to eliminate them is judged according to the actual clinical situation to ensure the integrity and accuracy of the data.

## 3 Results

### 3.1 Balance analysis of baseline data

**Figure 2:**
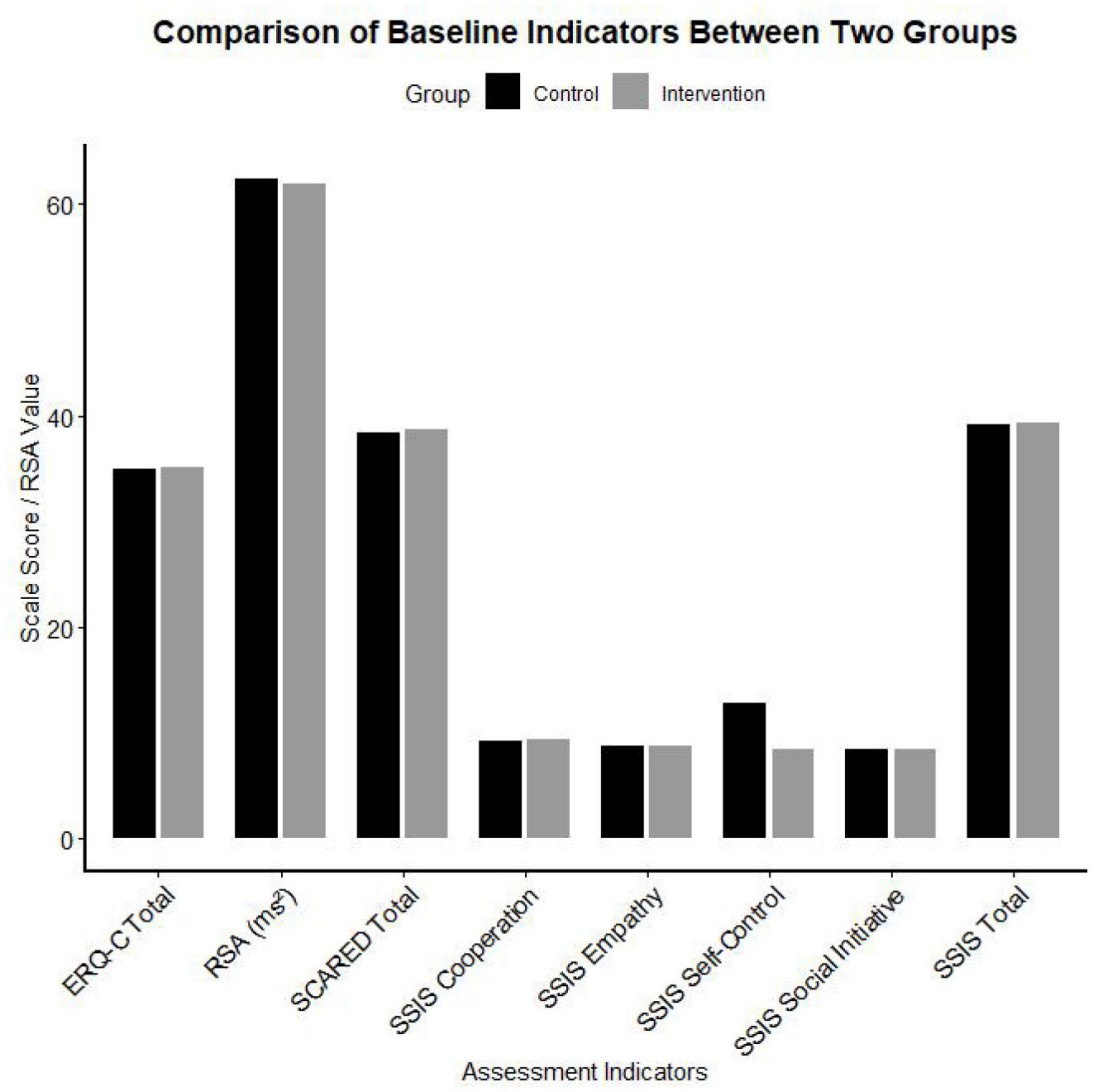
Comparison histogram of baseline observation indicators of two groups of children. *X*-axis represents assessment indicators; *Y*-axis denotes scale scores or RSA magnitude (ms^2^). Black bars = control group; gray bars = intervention group. No significant between-group differences were observed at baseline (*P* > 0.05), confirming balanced group demographics and clinical metrics.

This study enrolled a total of 120 trauma-exposed children aged 6–12 years who met the inclusion criteria and received treatment in the pediatric department of our hospital. Using a random number table, the participants were divided into an intervention group (60 participants) and a control group (60 participants). Initially, 132 participants were recruited; 12 dropped out during the study. The dropout stages and reasons are detailed in the CONSORT flowchart. Ultimately, 120 participants fully completed the 8-week intervention and 3-months follow-up, with no further dropouts. The two groups of children were tested for baseline balance in demographic sociology data, trauma characteristics data, scale scores and physiological indexes before intervention, and independent sample *t*-test was used for age, scale scores and RSA level. The statistical data of gender, trauma type, family structure and parents' education level were tested by χ^2^ test, and there was no statistical difference among all baseline indicators (*P* > 0.05). The baseline data of the two groups were well comparable and had a statistical basis for the comparison of follow-up intervention effects. The sample included in this study comprises four types of trauma: accidents, violent injuries, the death of a loved one, and sudden disasters. The psychological impacts of these different types of trauma vary, and subgroup analyses will be conducted for each type in the following sections (Tables 1, 2 and Figure 2).

**Table 1:** Comparison of sociological baseline data of general population of two groups of children.

Index	Classify	Control group (*n* = 60)	Intervention group (*n* = 60)	χ^2^/*t* value	*P*-value
Age (x̄ ±*s*, years old)	–	8.46 ± 1.32	8.39 ± 1.28	0.297	0.767
Gender	Man	33 (55.00%)	31 (51.67%)	0.135	0.713
	Woman	27 (45.00%)	29 (48.33%)		
Trauma exposure time (x̄ ±*s*, months)	–	3.52 ± 1.06	3.47 ± 1.12	0.251	0.802
Trauma type	Accident	26 (43.33%)	24 (40.00%)	0.308	0.958
	Violent injury	15 (25.00%)	16 (26.67%)		
	Death of a loved one	11 (18.33%)	12 (20.00%)		
	Sudden disaster	8 (13.33%)	8 (13.33%)		
Family structure	Nuclear family	42 (70.00%)	40 (66.67%)	0.154	0.695
	Single parent/reorganized family	18 (30.00%)	20 (33.33%)		
Father's educational level	Junior high school and below	22 (36.67%)	24 (40.00%)	0.271	0.873
	High school/technical secondary school	25 (41.67%)	23 (38.33%)		
	College or above	13 (21.67%)	13 (21.67%)		
Mother's educational level	Junior high school and below	20 (33.33%)	21 (35.00%)	0.186	0.911
	High school/technical secondary school	26 (43.33%)	24 (40.00%)		
	College or above	14 (23.33%)	15 (25.00%)		

**Table 2:** Baseline comparison of various scales and physiological indexes between the two groups before intervention (x̄ ± *s*).

Index	Control group (*n* = 60)	Intervention group (*n* = 60)	*T-*value	*P-*value
Total score of ERQ-C emotional regulation	34.96 ± 7.15	35.18 ± 7.24	0.168	0.867
Total score of SSIS social skills	39.15 ± 6.92	39.27 ± 6.89	0.095	0.924
SSIS cooperation dimension	9.26 ± 2.15	9.31 ± 2.08	0.130	0.897
SSIS empathy dimension	8.75 ± 1.96	8.69 ± 2.03	0.165	0.869
SSIS social initiative dimension	8.42 ± 2.23	8.37 ± 2.19	0.124	0.901
SSIS self-control dimension	12.72 ± 2.56	12.90 ± 2.48	0.392	0.696
SCARED total score of anxiety and depression	38.42 ± 8.36	38.65 ± 8.42	0.150	0.881
RSA level (ms^2^)	62.35 ± 9.46	61.89 ± 9.52	0.264	0.792

### 3.2 Comparison of ERQ-C scores between the two groups before and after intervention

**Figure 3:**
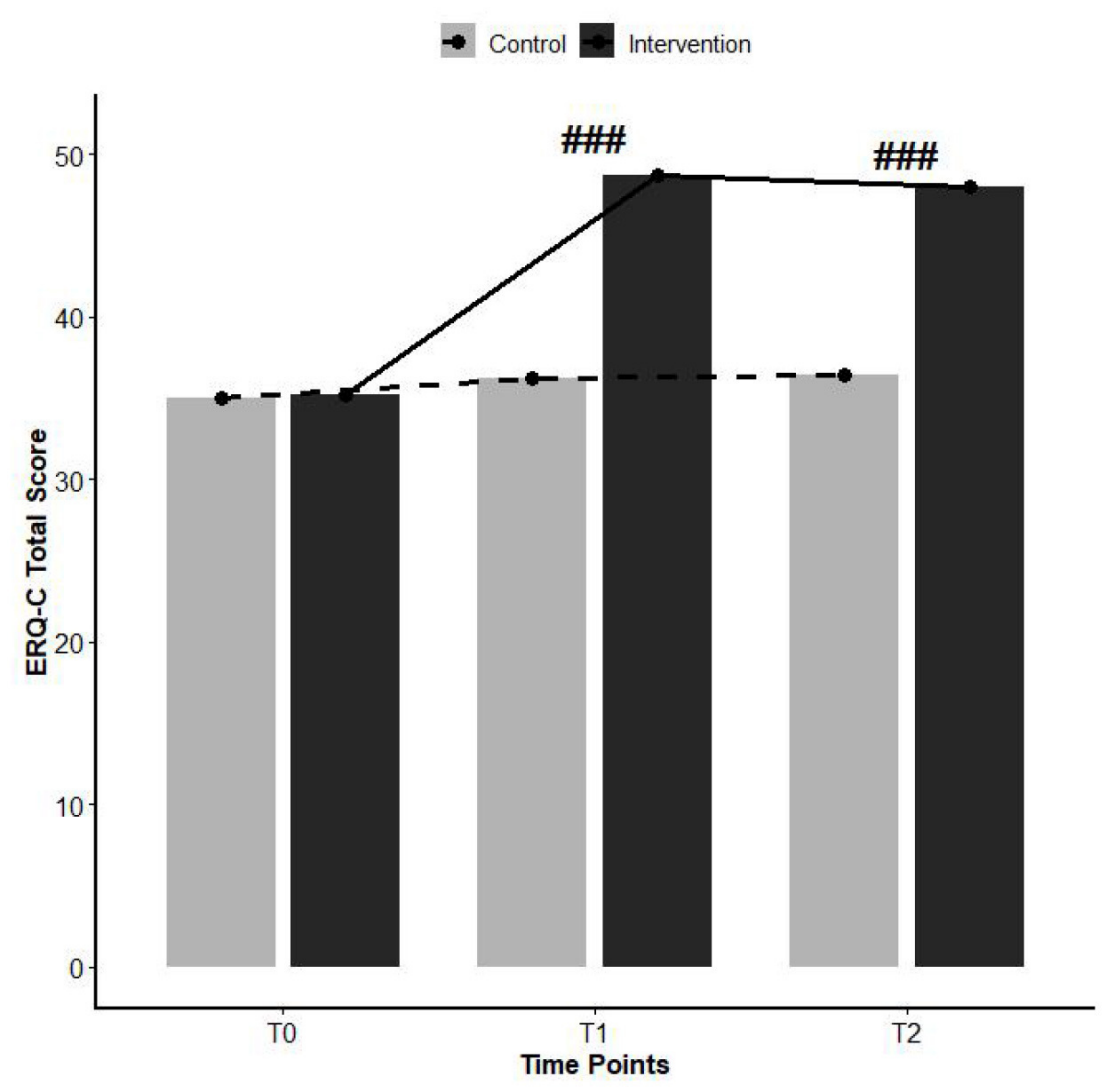
The changing trend of ERQ-C score in two groups of children at different time points. The abscissa is the evaluation time nodes T0, T1 and T2, and the ordinate is the total score of ERQ-C; The solid line is the intervention group and the dotted line is the control group; Control group scores demonstrated no meaningful temporal fluctuations, and the score of the intervention group increased significantly after intervention and remained high during the follow-up period, with significant differences between the groups.

Intra-group comparison: The total score of ERQ-C in the control group after intervention (T1) and follow-up for 3 months (T2) was not significantly improved compared with that before intervention (T0; *P* > 0.05). The total score of ERQ-C emotion regulation in the intervention group was significantly higher than that in T0 at T1 and T2, and between-group differences reached statistical significance at P < 0.001. Comparison between groups: There was no difference in the total score of ERQ-C between the two groups at time T0 (*P* > 0.05). The total score of ERQ-C in the intervention group was significantly higher than that in the control group at T1 and T2, and the difference was highly statistically significant (*P* < 0.001). At the 3-months follow-up, the effect size for the intervention group was Cohen's *d* = 0.89, indicating a large effect; at the group mean level, the intervention's effects were found to be stable and sustained (Table 3 and Figure 3).

**Table 3:** Comparison of the total score of ERQ-C emotion regulation between the two groups at different time points (x̄ ± s).

Group	Number of cases	T0 (before intervention)	T1 (after intervention)	T2 (follow-up for 3 months)	*F*-value	*P*-value
Control group	60	34.96 ± 7.15	36.25 ± 6.87	36.41 ± 7.02	0.872	0.419
Intervention group	60	35.18 ± 7.24	48.72 ± 6.35^###^	47.95 ± 6.58^###^	45.631	<0.001
*T-*value	–	0.168	10.352	9.476	–	–
*P-*value	–	0.867	<0.001	<0.001	–	–

Compared with T0 in the same group, ^###^*P* < 0.001. Multiple comparisons have been corrected, and 95% CIs have been calculated for all results.

### 3.3 Comparison of the total score and each dimension score of SSIS between the two groups before and after intervention

Intra-group comparison: The total score of social skills and scores of cooperation, empathy, social initiative and self-control of SSIS in the control group at time points T1 and T2 were not statistically different from those of T0 (*P* > 0.05). The total score of SSIS in T1 and T2 in the intervention group was significantly higher than that in T0 (*P* < 0.01 or *P* < 0.001), among which the dimensions of cooperation, empathy and social initiative were the most prominent. Comparison between groups: At T0, the total score of SSIS and the scores of each dimension were balanced and comparable (^*^P > 0.05); The total score of SSIS and the scores of all dimensions in T1 and T2 intervention groups were significantly higher than those in the control group (*P* < 0.001), and the overall social adaptability was significantly improved. The effect of follow-up for 3 months was 0.78–0.92 (Table 4 and Figure 4).

**Table 4:** Comparison of the total score and dimension score of SSIS social skills between the two groups at different time points (x̄ ± *s*).

Index	Group	T0	T1	T2
SSIS total score	Control group	39.15 ± 6.92	40.32 ± 7.11	40.56 ± 6.98
	Intervention group	39.27 ± 6.89	52.41 ± 7.03^###&^	51.86 ± 6.75^###&^
Cooperation dimension	Control group	9.26 ± 2.15	9.51 ± 2.08	9.48 ± 2.12
	Intervention group	9.31 ± 2.08	13.65 ± 2.34^###&^	13.42 ± 2.26^###&^
Empathy dimension	Control group	8.75 ± 1.96	8.92 ± 1.87	8.89 ± 1.91
	Intervention group	8.69 ± 2.03	12.84 ± 2.15^###&^	12.67 ± 2.09^###&^
Social initiative	Control group	8.42 ± 2.23	8.65 ± 2.16	8.71 ± 2.20
	Intervention group	8.37 ± 2.19	12.53 ± 2.41^###&^	12.38 ± 2.35^###&^
Self-control	Control group	12.72 ± 2.56	13.24 ± 2.49	13.38 ± 2.53
	Intervention group	12.90 ± 2.48	13.98 ± 2.36^##&^	13.85 ± 2.42^##&^

## *P* < 0.01, ^###^ *P* < 0.001 compared with the same group T0; compared with the control group at the same time point, &*P* < 0.001. Multiple comparison correction was performed, and 95% confidence intervals (95% *CI*) were presented in tables and figures.

**Figure 4:**
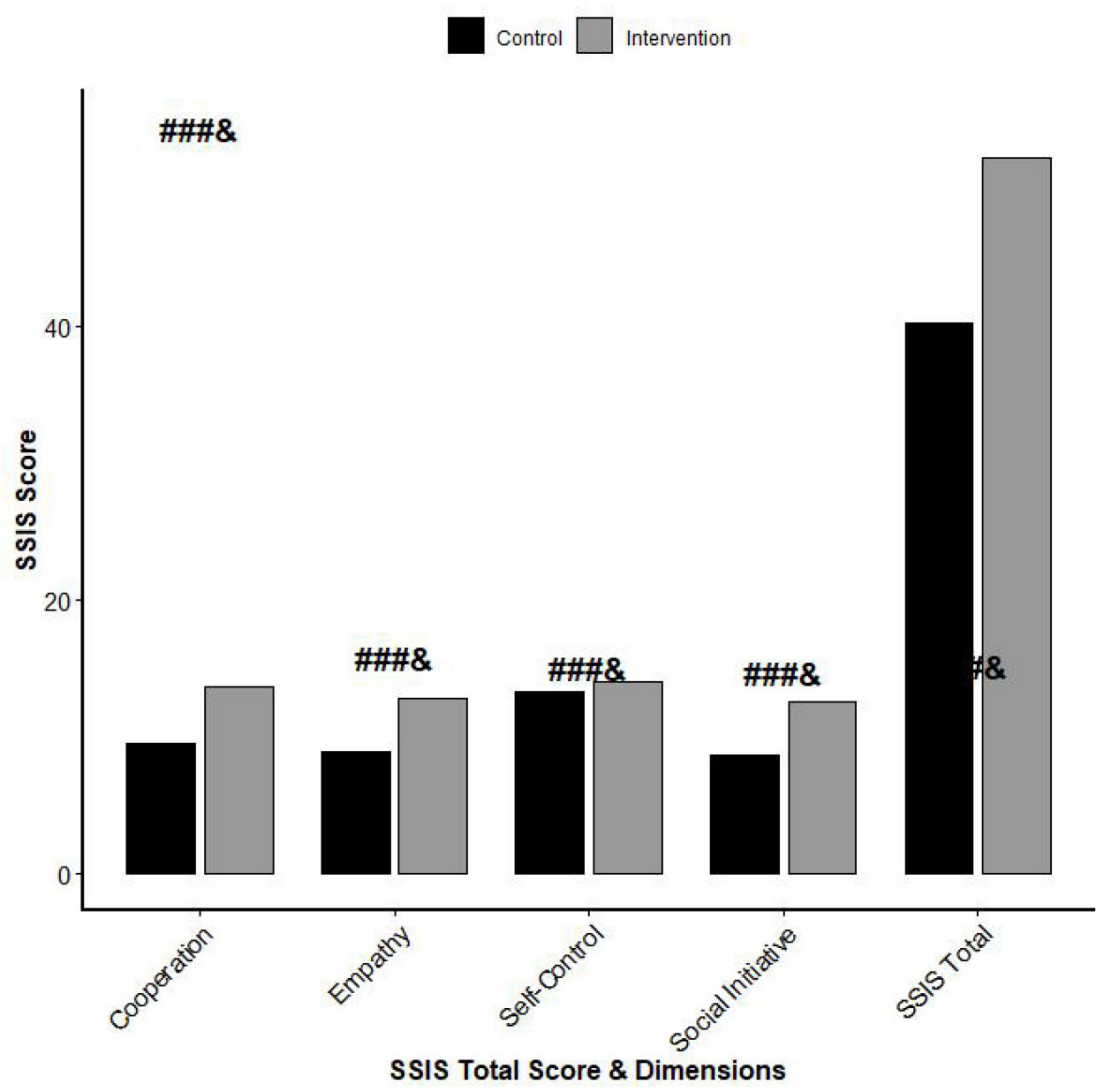
Comparison histogram of SSIS total score and dimensions between two groups after intervention. The abscissa is the total score and four dimensions of SSIS, and the ordinate is the scale score; Black column control group, gray column intervention group; All the indexes in the intervention group were significantly higher than those in the control group, and the dimensions of cooperation, empathy and social initiative improved more significantly.

### 3.4 Comparison of scores of children's anxiety and depression symptoms (SCARED) between the two groups before and after intervention

**Figure 5:**
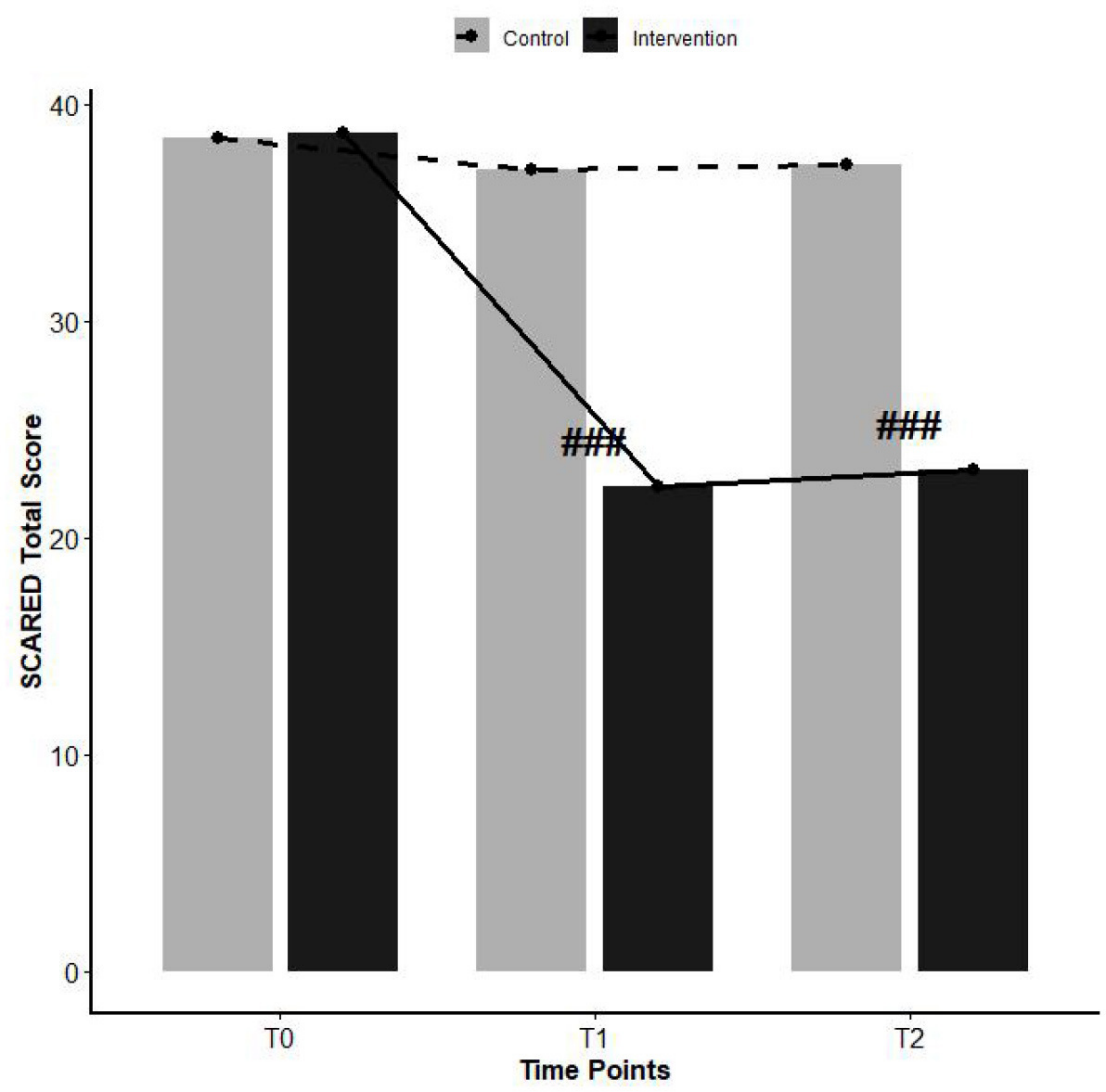
Time trend chart of SCARED score in two groups of children. The abscissa is T0, T1 and T2, and the ordinate is the total score of SCARED; The score of the control group fluctuated slightly, and the score of the intervention group decreased significantly after intervention, and the follow-up effect was stable.

Intra-group comparison: Scared scores of T1 and T2 in control group were not significantly lower than that of T0 (*P* > 0.05); In the intervention group, the total score of anxiety and depression in T1 and T2 SCARED was significantly lower than that in T0 (*P* < 0.001), and the internalized emotional symptoms were obviously relieved. Comparison between groups: There was no difference in SCARED score between the two groups at T0 (^*^P^*^ > 0.05); SCARED score of T1 and T2 intervention group was significantly lower than that of control group (*P* < 0.001), and it remained low after 3 months of follow-up, with the effect of 0.92 (Table 5 and Figure 5).

**Table 5:** Comparison of the total SCARED scores between the two groups at different time points (x̄ ± *s*).

Group	Number of cases	T0	T1	T2
Control group	60	38.42 ± 8.36	36.95 ± 7.84	37.21 ± 8.15
Intervention group	60	38.65 ± 8.42	22.36 ± 5.17^###^	23.15 ± 5.42^###^
*T*-value	–	0.150	12.065	11.328
*P*-value	–	0.881	<0.001	<0.001

Compared with T0 in the same group, ^###^*P* < 0.001. Multiple comparison correction was performed, and 95% confidence intervals (95% *CI*) were presented in tables and figures.

### 3.5 Comparison of RSA level of autonomic nervous function between two groups before and after intervention

**Figure 6:**
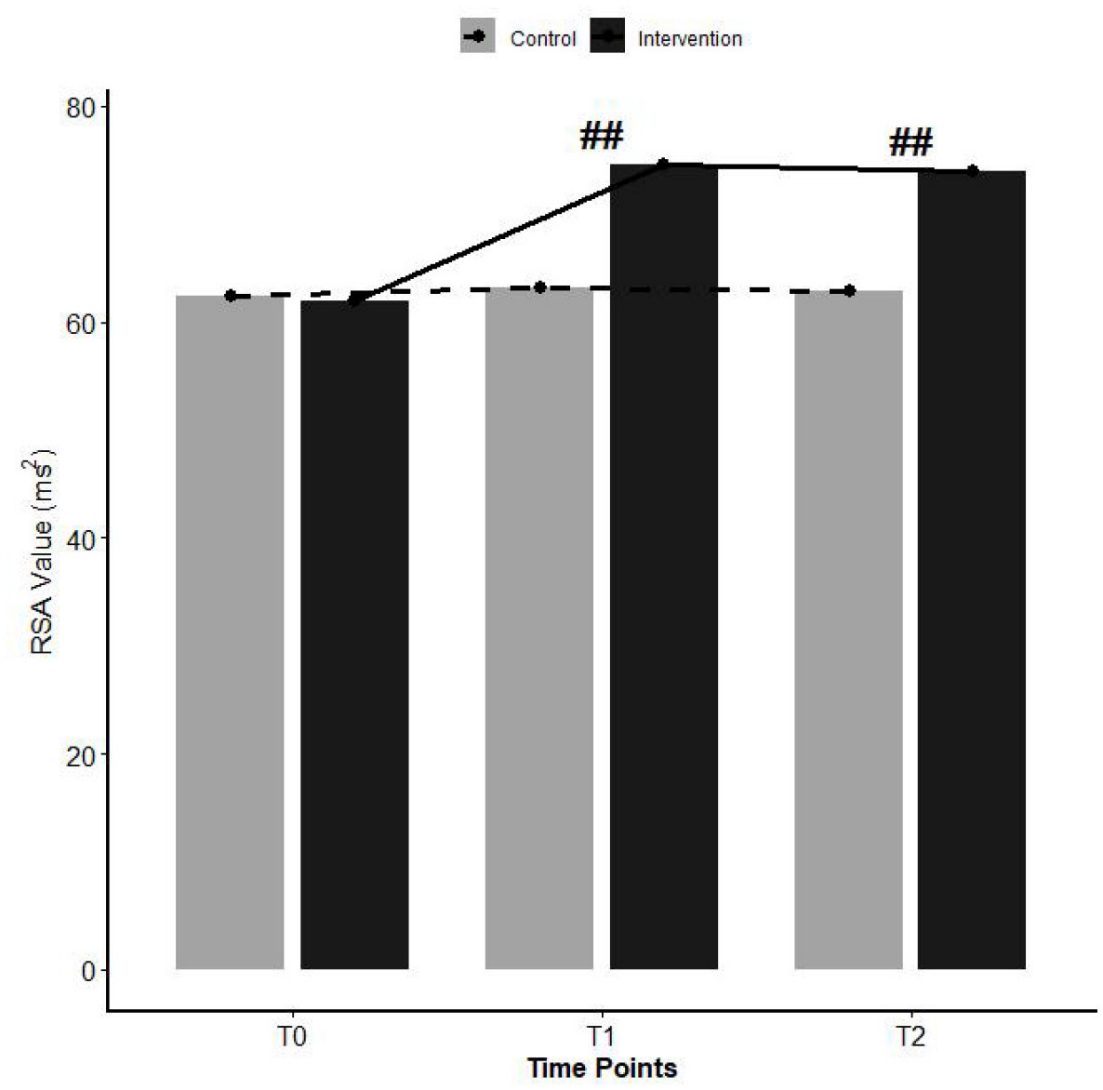
histogram of RSA level before and after intervention in two groups of children. The abscissa is T0, T1 and T2, and the ordinate is RSA value (ms^2^); The RSA level in the intervention group increased significantly after intervention, but there was no significant change in the control group, and the difference between the two groups was statistically significant.

Intra-group comparison: There was no significant change in RSA level in the control group at each time point (*P* > 0.05). The RSA levels of T1 and T2 in the intervention group were significantly higher than those of T0 (*P* < 0.01), suggesting that parasympathetic nervous system activity was up-regulated and the physiological basis of autonomic nerve emotion regulation was improved. Comparison between groups: At T0, the baseline of RSA level in the two groups was consistent (^*^P^*^ > 0.05); The RSA level in T1 and T2 intervention group was significantly higher than that in control group (*P* < 0.01) (Table 6 and Figure 6).

**Table 6:** Comparison of RSA level between two groups at different time points (x̄ ± *s*, ms^2^).

Group	Number of cases	T0	T1	T2
Control group	60	62.35 ± 9.46	63.12 ± 9.25	62.87 ± 9.34
Intervention group	60	61.89 ± 9.52	74.56 ± 8.93^##^	73.92 ± 9.05^##^
*T*-value	–	0.264	6.892	6.457
*P*-value	–	0.792	<0.01	<0.01

Compared with T0 in the same group, ^##^*P* < 0.01. Multiple comparison correction was performed, and 95% confidence intervals (95% *CI*) were presented in tables and figures.

### 3.6 Analysis of intervention safety and follow-up compliance

All 120 eligible participants in this study completed the 8-week intervention and the 3-months follow-up. The overall adherence rate to the intervention in the intervention group was 96.25%, and both the number of participants who received make-up sessions and the number of make-up sessions met the predefined criteria. During the intervention period, only 2 children had temporary emotional irritability, which was quickly relieved after immediate appeasement, and there were no serious adverse reactions such as headache, nausea and persistent crying. During the follow-up period, there were no long-term adverse events related to trauma-informed music intervention, and the intervention safety was good.

### 3.7 Analysis of intervention effect

**Figure 7:**
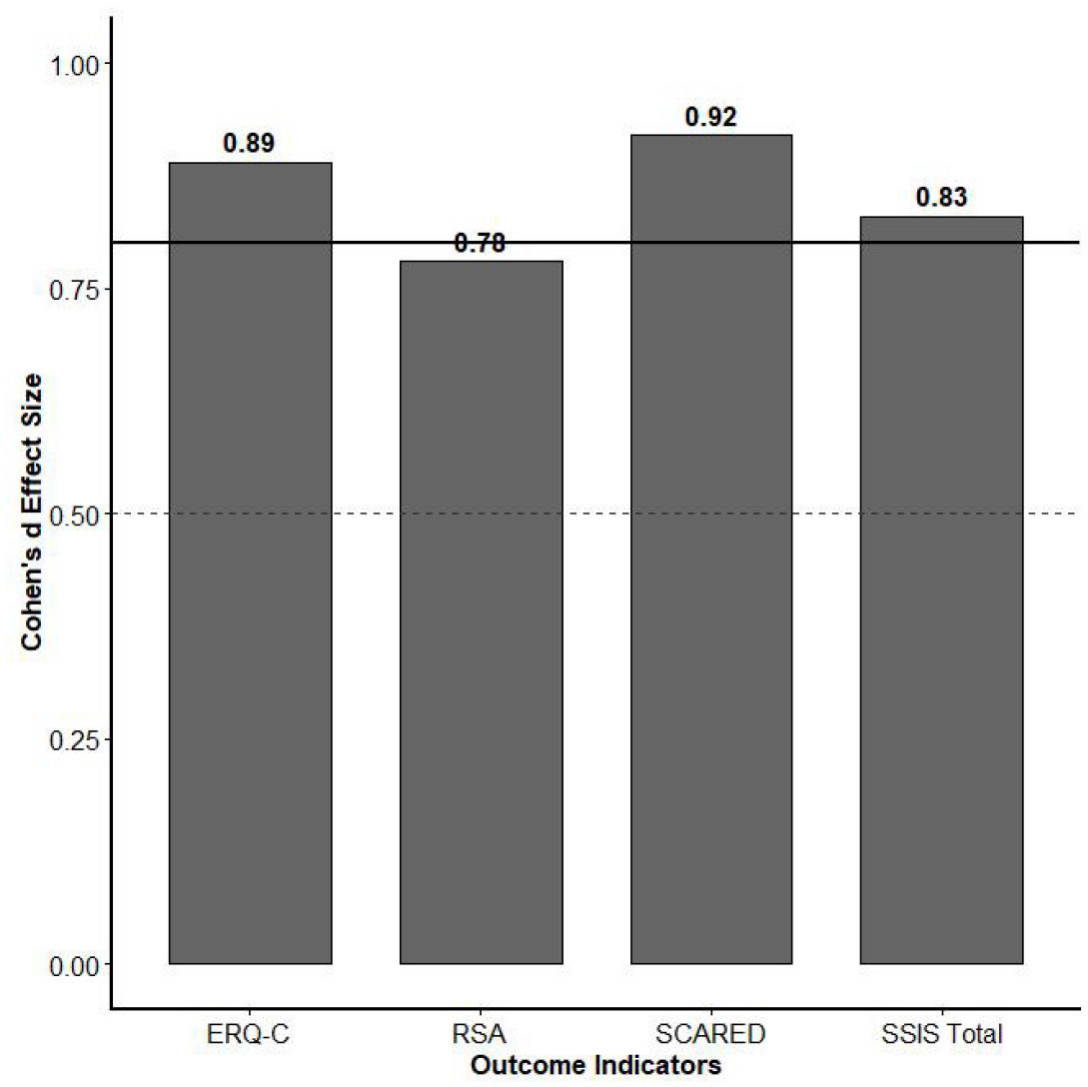
Cohen's *d* effect sizes of intervention outcomes.

Effect size for the primary outcome measure at 3 months of follow-up, calculated using Cohen's *d* (based on the combined standard deviation of the two groups): ERQ-C was 0.89, SSIS was 0.83, SCARED was 0.92 and RSA was 0.78; SCARED and ERQ-C are big effects, while RSA and SSIS are above average effects, suggesting that trauma-informed music intervention has a stable and considerable clinical effect on emotional adjustment, social adaptation and improvement of anxiety and depression in children with pediatric trauma exposure (Figure 7).

## 4 Discussion

### 4.1 The mechanism of trauma-informed music intervention to improve emotional regulation and internalization symptoms of traumatized children

Exposure to trauma in childhood can lead to dysfunction in the prefrontal cortex–amygdala–hippocampus neural circuit and excessive activation of the HPA axis, manifesting as impaired emotional regulation, amplified negative emotions, and internalized symptoms such as anxiety and depression—which are core characteristics of post-traumatic psychological impairment (11). This study showed that the intervention group's total ERQ-C emotional regulation score increased by 13.54 points from baseline, and their SCARED total scores decreased by 16.29 points, with effect sizes of 0.89 and 0.92, respectively. Although intergroup differences reached statistical significance, the biological mechanisms driving these improvements remain only theoretical speculation. This study did not conduct direct assessments such as neuroimaging, cortisol levels, or neurotransmitter measurements, consistent with conclusions proposed by other researchers (12). Current theoretical hypotheses suggest that musical stimulation may act on the brain's limbic system to reduce excessive excitability in the amygdala, while simultaneously enhancing the prefrontal cortex's ability to regulate emotions and promoting the formation of cognitive reappraisal strategies (13); structured modules such as rhythmic patterns and improvisation are hypothesized to induce the brainstem to release neurotransmitters such as 5-serotonin and dopamine, among other neurotransmitters, thereby modulating HPA axis activity, lowering cortisol levels, and alleviating stress responses (14). Trauma-informed music intervention creates a safe, non-judgmental space for expression, bypassing language barriers to allow children to release repressed emotions through music, reduce expressive inhibition, and promote emotional integration on a psychological level (15). This study also found that RSA levels in the intervention group increased significantly. Since RSA is susceptible to interference from various factors-such as respiratory status, momentary emotions, and the external environment-a single RSA indicator cannot fully reflect the neurophysiological mechanisms of autonomic and emotional regulation and should only be used as a reference indicator. This result preliminarily aligns with theoretical hypotheses regarding “music-autonomic regulation” (16).

### 4.2 The value of trauma-informed music intervention to improve the social adaptability of traumatized children

Post-traumatic social avoidance, deficits in empathy, and reduced cooperative abilities are the primary manifestations of social maladjustment in children, severely impacting the development of their interpersonal relationships and social functioning (17). In this study, the intervention group's total SSIS social skills score increased by 13.14 points, with particularly notable improvements in the dimensions of cooperation, empathy, and social initiative. The effect sizes at the 3-months follow-up ranged from 0.78 to 0.85. As this scale relies on subjective ratings by caregivers, reporting bias may be present. Additionally, the intervention group received more interpersonal interactions, group companionship, and therapist attention; these confounding factors may also have contributed to the improvement in social-related scores, consistent with findings from studies involving traumatized children (18). Group music activities (group ensemble playing and instrumental duets) provide children with low-stress social settings. Through interactive forms such as taking turns playing and coordinating rhythms, these activities gradually foster eye contact, empathetic responses, and a sense of collaboration, thereby reducing social avoidance behaviors (19). During the process of creating emotional songs, children enhance their social initiative and communication skills by collectively discussing lyrics and adapting melodies, thereby rebuilding their social confidence (20). Compared to traditional psychological interventions, trauma-informed music interventions-which center on non-verbal interaction-are better suited to the characteristics of traumatized children, such as limited verbal expression and social anxiety. They can effectively overcome social barriers and promote the restoration of social adaptive functioning (21). However, this study was unable to distinguish the independent effects of music itself, the additional attention provided, and group activities.

### 4.3 Safety, compliance and application advantages of trauma-informed music intervention

In this study, there were no cases falling off, and the compliance rate of intervention was 100%. Only two cases experienced short-term emotional irritability and quickly relieved, and there were no serious adverse reactions, which confirmed that trauma-informed music intervention had high safety and high acceptance, which was consistent with the research conclusion (22). Traditional traumatic psychological intervention (TF-CBT, EMDR) has some limitations, such as long implementation period, shortage of professionals, high requirements for language ability, and children's compliance is generally low (23). Trauma-informed music intervention is simple and interesting, which stimulates children's participation motivation through musical instrument exploration, rhythm games and other forms, without relying on language expression, and adapts to the cognitive and psychological development characteristics of children aged 6–12 (24); The requirement of intervention site is low, and it can be implemented by music therapists with standardized training, which is convenient for clinical popularization and application (25). In this study, an 8-week structured intervention program was adopted to promote relationship building, emotional expression and skill integration in stages, ensuring the systematicness and effectiveness of the intervention, which provided a reference for the standardization of clinical intervention programs (26).

### 4.4 Research limitations and future research direction

This study has several limitations: This single-center trial limits the external validity of findings, which cannot be generalized to clinical populations or medical facilities in distinct geographic regions (27). The absence of an active control group makes it impossible to rule out confounding factors such as additional interpersonal attention and group activities; reliance solely on a single RSA physiological indicator and a parent-rated questionnaire introduces significant subjective bias and highlights the limitations of these measures (28); the follow-up period was relatively short, and long-term efficacy remains unknown; the neuro- and endocrine-related mechanisms of action are theoretical hypotheses that have not been confirmed by direct data; and the limited scope of sample inclusion makes it difficult to generalize the results to populations of traumatized children with comorbid mental disorders (29).

### 4.5 Clinical practice enlightenment

This study confirms that trauma-informed music intervention shows promise as a preferred non-pharmacological intervention for the psychological rehabilitation of traumatized children, particularly those with limited verbal expression, severe emotional disturbances, and social withdrawal. In clinical practice, it can be incorporated into the standard psychological rehabilitation system for pediatric trauma, used in conjunction with routine psychological support to enhance intervention outcomes; A standardized intervention manual should be developed to strengthen collaboration among music therapists, pediatricians, and psychotherapists, thereby promoting a multidisciplinary intervention model; intervention modules should be tailored to children of different ages and with different types of trauma. In clinical application, the results of this study should be interpreted rationally, distinguishing between statistical differences and actual clinical benefits, while also emphasizing the added value of interpersonal companionship and group interaction.

## 5 Conclusion

This study preliminarily explored the effects of an 8-week trauma-informed music intervention on children aged 6–12 who had been exposed to trauma. Statistical comparisons between groups showed that the trauma-informed music intervention combined with routine psychological support, compared to routine psychological support alone, led to higher scores on the Emotional Regulation Scale and Social Skills Scale, lower scores on the Anxiety and Depression Scale, and an upward trend in RSA levels. Changes in these indicators remained relatively stable during the short-term follow-up period; the intervention was well-tolerated and well-received by the children. Trauma-informed music intervention can serve as an adjunctive treatment for the psychological rehabilitation of school-aged children exposed to trauma and warrants further in-depth research and clinical exploration. Future studies should optimize the research design, refine the assessment system, extend the follow-up period, and conduct higher-quality clinical trials to validate its actual efficacy and mechanisms of action.

## Data Availability

The raw data supporting the conclusions of this article will be made available by the authors, without undue reservation.
